# Short‐Term Outcomes of Full Pulpotomy Compared With Root Canal Treatment for Irreversible Pulpitis: The PROVE Study

**DOI:** 10.1111/iej.70208

**Published:** 2026-06-29

**Authors:** I. A. El Karim, H. F. Duncan, S. G. Craig, V. Ballal, R. Narkedamalli, N. A. Taha, R. H. Albanna, P. Chompu‐inwai, T. Srisuwan, V. Yu, S. Chua, S. Saber, K. R. Foo, H. M. A. Ahmed, A. Salami, S. Cushley, M. Clarke

**Affiliations:** ^1^ School of Medicine, Dentistry and Biomedical Sciences Queen's University Belfast Belfast UK; ^2^ Dublin Dental University Hospital, Trinity College Dublin Dublin 2 Ireland; ^3^ Department of Conservative Dentistry and Endodontics Manipal College of Dental Sciences Manipal Academy of Higher Education Manipal India; ^4^ Department of Conservative Dentistry Jordan University of Science and Technology Irbid Jordan; ^5^ Faculty of Dentistry Chiang Mai University Chiang Mai Thailand; ^6^ Faculty of Dentistry National University of Singapore Singapore Singapore; ^7^ Faculty of Dentistry The British University in Egypt (BUE) Elsherouk Egypt; ^8^ Faculty of Dentistry University Malaya Kuala Lumpur Malaysia; ^9^ Mohammed Bin Rashid University of Medicine and Health Sciences, Dubai Health Dubai UAE

**Keywords:** failure, outcomes, pain, vital pulp treatment

## Abstract

**Objectives:**

To assimilate data from individual randomised trials and perform a pooled analysis of short‐term outcomes of pulpotomy compared with root canal treatment (RCT) for teeth with symptomatic irreversible pulpitis (SIRP).

**Methods:**

Randomised trials were conducted in eight countries using a standardised clinical protocol and outcome measures. Inclusion criteria were a diagnosis of SIRP in a restorable tooth that could be adequately isolated. A block randomisation method was used. Short‐term outcomes were postoperative pain (assessed at baseline, day 3 and day 7), analgesic consumption and early failure, which was defined as the need for additional intervention within 4 weeks of treatment. Data were analysed using R v4.3.1.

**Results:**

A total of 410 patients were randomised, with data available for 385 patients. A significant reduction in pain intensity was observed over time for each treatment group (*p* < 0.0001). Participants who received RCT were observed to have a significantly greater decrease in pain intensity between baseline and day 3, mean Numeric Rating Scale (NRS‐11) of 1.7 (*p* = 0.0034) and from day 3 to day 7 with a mean NRS of 0.7 (*p* = 0.0471) compared with participants treated with pulpotomy who had mean NRS of 2.4 and 1.2 respectively. Use of analgesics remained high between baseline and day 3 for participants treated with pulpotomy but significantly decreased for RCT (51.1%, 70/137 vs. 33.3%, 46/138; *p* = 0.0042). The use of analgesics was significantly reduced in both treatment groups between day 3 and day 7 but was significantly higher at day 7 for participants treated with pulpotomy compared to RCT (19.7%, 27/137 vs. 9.4%, 13/138; *p* = 0.0246). Early failure was significantly more common for participants who underwent pulpotomy treatment (10/192; 5.2%) compared with RCT (1/193; 0.5%) (*p* = 0.0141) and was associated with preoperative tenderness to percussion (TTP), molar teeth, sleep disturbance and prolonged bleeding time after exposure.

**Conclusion:**

The PROVE study provides the first robust evidence that both pulpotomy and RCT provided effective pain relief up to day 7; but significantly more pain relief and reduced analgesic intake were evident after RCT. Early failure rate was low in both groups.

## 1 Introduction

Untreated dental caries remains the most common non‐communicable chronic disease worldwide and the leading cause for patients to seek dental treatment for pain relief (Rechenberg et al. 2016; Kassebaum et al. 2015). Carious bacteria and their by‐products diffuse into the dental pulp causing inflammation and pain. This is clinically described as symptomatic irreversible pulpitis (SIRP) (Glickman 2009). Traditionally SIRP is treated by either extraction or root canal treatment (RCT), however, an improved understanding of pulp biology has improved clinicians' understanding of the biological and reparative processes in the injured dental pulp (Duncan, Cooper, and Smith 2019; El karim et al. 2021) which coupled with introduction of bioactive hydraulic calcium silicate cements (Nair et al. 2009; Parirokh et al. 2018) has promoted pulpotomy as a biologically‐based treatment alternative for SIRP. Consequently, many clinical studies reporting on the outcome of pulpotomy in teeth with signs and symptoms indicative of SIRP have been published, demonstrating high success rates (Taha and Abdelkhader 2018; Asgary et al. 2013; Cushley et al. 2019). With this upsurge in interest in vital pulp treatment (VPT), the European Society of Endodontology (ESE) and the American Association of Endodontists (AAE) developed position statements to recommend pulp preservation therapies, such as pulpotomy, for managing SIRP, while emphasising the limited evidence and need for well‐designed and adequately powered trials to determine the clinical effectiveness of pulpotomy as an alternative to RCT (Duncan, Galler, et al. 2019; AAE Position Statement on Vital Pulp Therapy 2021). In the recently published ESE‐level S3 clinical practice guidelines, pulpotomy was suggested as an alternative to RCT but the evidence for clear differences in patient‐reported pain at day 7 postoperatively, long‐term clinical success and effectiveness between RCT and pulpotomy has been reported as being limited (Tomson et al. 2023; Duncan et al. 2023; Taha et al. 2023). Pain relief is considered the most important patient reported outcome following vital pulp treatment (Cushley et al. 2022; Duncan et al. 2021; El Karim et al. 2024) and further evidence on effective postoperative pain relief is warranted.

The robust effectiveness trials required for guideline development and subsequent change in clinical practice requires a high‐power, generalisable and multicentre approach, with associated financial and logistical implications. To overcome these challenges, a series of linked randomised trials following a standardised published protocol was suggested as a method that could offer the advantages of efficient adaptive design, the ability to study low failure levels and increase external validity of results (Tierney et al. 2015). On this basis, an international VPT consortium was established to undertake a series of individual but linked randomised trials to investigate the clinical and cost‐effectiveness of pulpotomy compared with RCT. The PROVE study ‘Effectiveness of Full Pulpotomy Compared with Root Canal Treatment in Managing Teeth with Signs and SymptOms Indicative of Irreversible Pulpitis: A ProspectiVE Meta‐analysis of Individual Participant Data’, was designed to assess several clinical and non‐clinical outcomes at short and long‐term follow‐up periods, as described in the published protocol (El Karim et al. 2023). By applying unified protocols and outcome measures, PROVE aims to provide unequivocal evidence on the clinical and cost effectiveness of pulpotomy compared with RCT for teeth with signs and symptoms indicative of SIRP.

This first report of the PROVE study focuses on the secondary short‐term outcomes of the interventions, mainly post‐operative pain relief and early failure. The underlying hypothesis is that caries‐induced inflammation is often confined to the coronal pulp (Ricucci et al. 2014) and that removal of the coronal pulp by full pulpotomy may provide pain relief comparable to that achieved with pulpectomy. The following objectives will be addressed: (1) Assess the degree of postoperative pain relief in pulpotomy compared with RCT at day 3 and day 7 post‐intervention, (2) Assess the need for postoperative analgesic intake at days 3 and 7 following pulpotomy and RCT, (3) Determine the incidence of early failure with pulpotomy and RCT and investigate the associated factors.

## 2 Methods

### 2.1 Study Design

A series of linked non‐inferiority, parallel group pragmatic randomised trials in which patients were randomly allocated to complete pulpotomy (intervention arm) or RCT (control arm).

### 2.2 Trial Sites

Randomised trials were conducted in 10 participating countries, with eight sites contributing data to this report: the British University in Egypt; Manipal College of Dental Sciences, India; Jordan University of Science and Technology, Jordan; Universiti Malaya, Malaysia; Queen's University, Belfast, Northern Ireland; National University of Singapore, Singapore; Chiang Mai University Thailand; and Mohammed Bin Rashid University of Medicine and Health Sciences, Dubai Health, UAE. Two sites, one in the USA and one in Brazil, were unable to provide sufficient data to be included in the analysis. Sites were recruited based on expertise in this area and previous experience of running randomised trials, with each site committing to the randomisation of 50 to 60 patients and the provision of data on the primary and secondary outcomes.

### 2.3 Ethics

Ethical approvals were obtained from respective ethical committees at all institutions and informed written consent was obtained from participants.

### 2.4 Inclusion Criteria

Patients aged 12 years or older with a mature permanent tooth demonstrating radiographic evidence of deep caries deep (i.e., caries extending to the inner quarter of dentine but will well defined radiopaque zone of dentine), extremely deep caries (i.e., caries extending through the full thickness of dentine) (ESE 2019) or deep restoration and signs/symptoms indicative of SIRP (moderate to severe spontaneous pain). The tooth needed to be responsive to sensibility testing, restorable and able to be adequately isolated during treatment. Only one posterior tooth (molar or premolar) per patient could be included.

### 2.5 Exclusion Criteria

Teeth with active periodontal disease (pocket depth > 5 mm); teeth indicated for elective RCT for restorative purposes; teeth with signs and symptoms indicative of apical periodontitis evident by presence of apical radiolucency (preoperative tenderness to percussion (TTP) was interpreted as a clinical sign reflecting the spread of pulpal inflammation to the surrounding periodontal tissues, rather than established apical periodontitis); patients with complex medical histories that may affect their caries' experience and healing ability (e.g., immunocompromised, radiotherapy); patients who were unable to consent; history of previous trauma to the tooth; and patients who are pregnant or breast‐feeding. Intraoperatively, patients with any evidence of purulence or excessive bleeding that cannot be controlled with a cotton pellet with 2%–4% sodium hypochlorite for 10 min were excluded.

### 2.6 Power and Sample Size Calculation

The PROVE study sample size was estimated for a non‐inferiority trial based on the prespecified binary primary outcome assessed at 12 months assuming no difference between the success rate of pulpotomy and RCT (90%) with a non‐inferiority margin of 10%. For such a trial, a total of 380 patients were required, if randomised on a 1:1 basis with 90% power to detect a non‐inferiority margin of 10% at a 5% significance level. The secondary short‐term outcomes were not included in the original sample size estimation and their analyses are presented as secondary and exploratory.

### 2.7 Patient Enrolment and Randomisation

Patients attending with signs and symptoms suggestive of SIRP were screened for eligibility. Diagnosis of SIRP was based on the AAE classification (Glickman 2009). Preoperative pain level was recorded using Numerical Rating Scale (NRS‐11), an 11‐point numeric scale with 0 representing ‘no pain’ and 10 representing ‘pain as bad as you can imagine’. A sensibility cold test was performed and periapical radiographs clearly showing the depth of caries lesion/restoration and periapical area were obtained as part of the diagnostic procedure. Following clinical and radiographic assessment and confirmed diagnosis, patients who fulfilled the inclusion criteria and agreed to participate in the trial were randomised to one of the treatment groups. The unit of randomisation was the participant and randomisation was completed using a block randomisation system, with randomly permuted blocks of four and eight. Conceladed allocation was performed using sealed envelope method. Due to the nature of the intervention, it was not possible to blind the patients or the operators.

### 2.8 Clinical Procedure

#### 2.8.1 Root Canal Treatment (Control)

As the PROVE study included linked pragmatic trials, this procedure was carried out using the methods and materials that participating clinicians would normally use for performing RCT (El Karim et al. 2023). A contemporary RCT protocol in line with standards outlined in the AAE Treatments Standards (AAE 2023) was followed. As variations in RCT protocols would make it difficult to compare with pulpotomy, certain variables were standardised, including adequate anaesthesia, use of dental dam, irrigation protocol with 2%–4% sodium hypochlorite, intra canal medication with calcium hydroxide, working length with combined radiographs and apex locators, automated nickel‐titanium instrumentation to accompany hand instrumentation and preparation to apical size 2–3 larger than initial apical file to ensure proper cleaning and shaping. In line with the pragmatic nature of the trial, RCT was conducted in one or two treatment visits, as per local site protocols.

#### 2.8.2 Full Pulpotomy (Intervention)

Following adequate anaesthesia and isolation with dental dam, access to the pulp was gained after non‐selective caries removal and confirmation of pulp exposure to de‐roof the pulp chamber and excision of the entire coronal pulp. The pulp chamber was irrigated with 2%–4% sodium hypochlorite solution and resultant bleeding from the remaining pulp was controlled with a cotton pellet soaked in 2%–4% sodium hypochlorite solution for 2 min and then repeated up to five times if required. Following complete haemostasis, the pulp stump was covered with Biodentine according to manufacturer instructions (Septodont Ltd., Saint Maur des Fausse ´s, France) and the tooth permanently restored with a restoration if treatment was completed in a single visit or temporised with glass ionomer cement for the final restoration to be placed in a second visit if the operator opted for 2‐visit treatment.

If haemostasis at the pulp wound was not achieved within 10 min, pulpectomy and RCT were carried out and the patient was excluded from the trial. Similarly, if no bleeding was evident when the pulp was exposed (either the entire pulp or in one of the canals in multi‐rooted teeth), the pulp was considered non‐vital, RCT was carried out and the participant was excluded from the study.

### 2.9 Follow‐Up and Data Collection

Postoperative pain was recorded by patients on day 3 and 7, using the NRS‐11. Patients were instructed on how to use the NRS‐11 at home on days 3 and 7 after the procedure. Analgesic intake was reported by patients during the collection of the pain data.

No routine follow‐up was scheduled after 7 days, but all participants were advised to re‐attend if they experienced persistent and intolerable pain. Patients attending with pain or issues that required further intervention such as RCT or extraction up to 4 weeks post intervention were considered early failure. Covariates collected include (1) Preoperative factors (age, gender, tooth type (molar/premolar), number of tooth surfaces affected (occlusal proximal), nature and severity of pain, tenderness to percussion, caries depth) and (2) Intraoperative factors (Bleeding time, operator and dental dam use).

### 2.10 Data Analysis

Anonymised data were obtained for each participant included in each of the trials. Data were checked for quality and completeness by two independent assessors. Heterogeneity and inconsistency were expected to be minimal due to the harmonisation arising from the standardisation of practices and outcome measures. Data were analysed using R v4.3.1 with ggplot2 and base packages installed (*R Foundation for Statistical Computing*, Vienna, Austria). As the current report focuses exclusively on secondary outcomes and in line with CONSORT guidance for non‐inferiority trials (Piaggio et al. 2012), secondary short‐term outcomes were analysed using standard superiority comparisons. The small number of failures, together with the absence of failures in some randomised groups at some sites, precluded robust meta‐analysis or regression modelling. Therefore, a pooled analysis and unadjusted comparisons were undertaken (Justo et al. 2025; Peduzzi et al. 1996). Chi‐Square and Kruskal–Wallis Rank‐Sum tests were used to assess the association between categorical and continuous variables.

## 3 Results

A total of 410 participants who fulfilled the inclusion criteria were randomised within the PROVE study (Figure 1). Following randomisation, 22 participants did not receive the intervention (22/410; 5.4%) and 3 had incomplete data at baseline (3/410; 0.7%) (Figure 1). These 25 patients were excluded from the final analysis. In total, 385 participants (385/410; 93.9%) were suitable for the pooled analysis of early clinical outcomes presented here. These participants had been randomised to pulpotomy (*n* = 192; 49.9%). Short‐term outcome data were available for all 192 participants allocated to pulpotomy group (100%) and for 193 of the 196 allocated for RCT (98%). In line with the randomisation process, no significant differences were observed between clinical variables and treatment groups at baseline (Table 1). Missing data of associated clinical variables were shown in Table 1.

**FIGURE 1:**
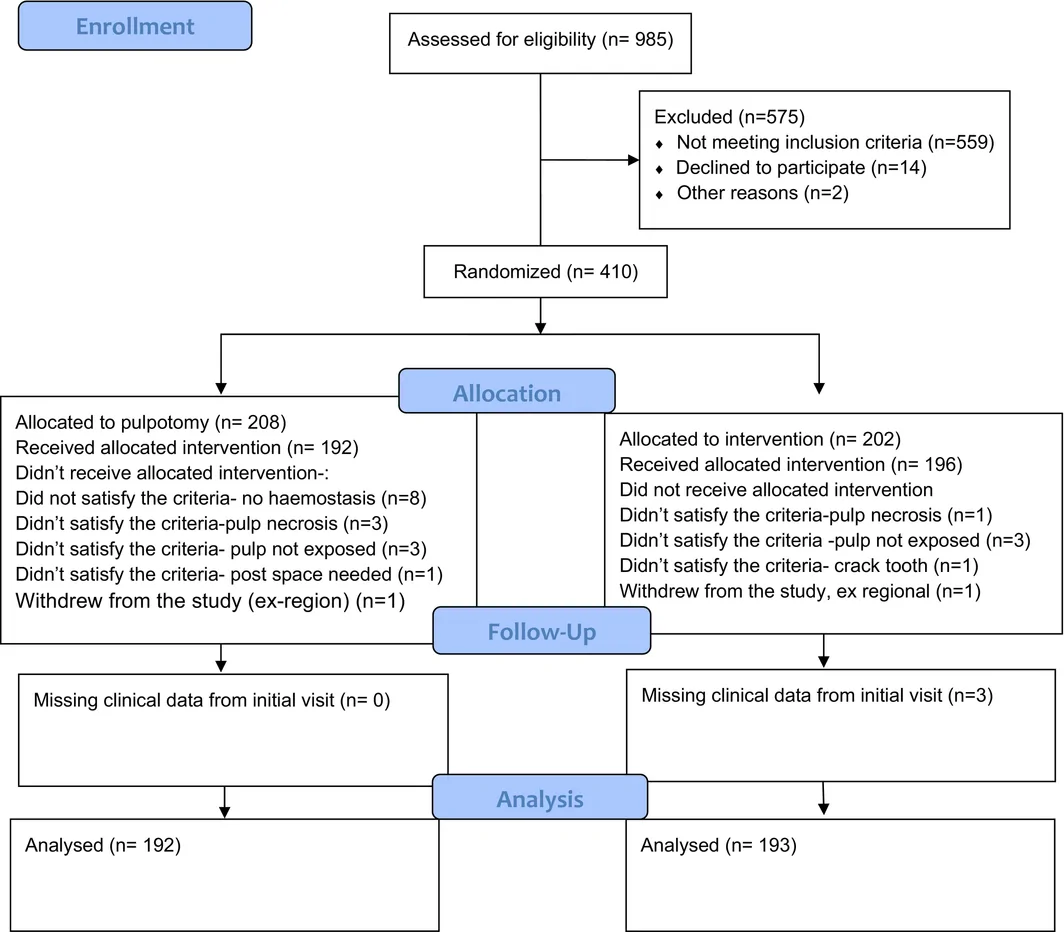
CONSORT diagram of PROVE participants for analyses of early clinical outcomes.

**TABLE 1:** Association of clinical variables of participants according to treatment received.

	Pulpotomy (*n* = 192)	Root canal treatment (*n* = 193)	*p*
Country			
Egypt	21 (10.9%)	24 (12.4%)	0.9945
India	30 (15.6%)	30 (15.5%)	
Jordan	27 (14.1%)	26 (13.5%)	
Malaysia	22 (11.5%)	20 (10.4%)	
Northern Ireland	30 (15.6%)	26 (13.5%)	
Singapore	18 (9.4%)	22 (11.4%)	
Thailand	24 (12.5%)	25 (13.0%)	
United Arab Emirates	20 (10.4%)	20 (10.4%)	
Gender			
Male	83 (43.2%)	89 (46.1%)	0.6407
Female	109 (56.8%)	104 (53.9%)	
Age	Range: 10–78, Median: 31	Range: 10–75, Median: 31	0.7617
Tooth number			
MO	154 (80.2%)	136 (70.5%)	0.0562
PR	37 (19.3%)	53 (27.5%)	
Missing	1 (0.5%)	4 (2.1%)	
Stimulated or spontaneous pain			
No Pain	6 (3.1%)	10 (5.2%)	0.1832
Spontaneous	97 (50.5%)	110 (57.0%)	
Stimulated	89 (46.4%)	73 (37.8%)	
Tenderness to percussion (TTP)			
0	130 (67.7%)	129 (66.8%)	0.5847
1	61 (31.8%)	64 (33.2%)	
Missing	1 (0.5%)	0 (0.0%)	
Use of analgesics			
No	83 (43.2%)	87 (45.1%)	0.6750
Yes	85 (44.3%)	81 (42.0%)	
Missing	24 (12.5%)	25 (13.0%)	
Sleep disturbance			
No	102 (53.1%)	108 (56.0%)	0.7426
Yes	88 (45.8%)	84 (43.5%)	
Missing	2 (1.0%)	1 (0.5%)	
Caries depth			
Deep	51 (26.6%)	43 (22.3%)	0.3557
Extremely deep	93 (48.4%)	111 (57.5%)	
None	2 (1.0%)	2 (1.0%)	
Missing	46 (24%)	37 (19.2%)	
Number of tooth surfaces affected			
1	30 (15.6%)	30 (15.5%)	0.5478
2	111 (57.8%)	109 (56.5%)	
3	16 (8.3%)	17 (8.8%)	
4	0 (0.0%)	3 (1.6%)	
Missing	35 (18.2%)	34 (17.6%)	
Dental dam used			
No	7 (3.6%)	11 (5.7%)	0.3873
Yes	184 (95.8%)	182 (94.3%)	
Missing	1 (0.5%)	0 (0.0%)	
Operator			
Endodontist	55 (28.6%)	62 (32.1%)	0.2666
General dental practitioner	39 (20.3%)	23 (11.9%)	
Paediatric dentist	5 (2.6%)	5 (2.6%)	
Post‐graduate dentist	70 (36.5%)	75 (38.9%)	
Missing	23 (12%)	28 (14.5%)	

*Note: p*‐values based on chi‐squared tests for association for categorical variables and the Mann–Whitney *U* test for continuous variables.

Abbreviations: MO, molar; PR, premolar.

### 3.1 Postoperative Pain Relief in Pulpotomy Compared With Root Canal Treatment at Day 3 and Day 7

A significant reduction in pain intensity was observed over time, as measured by the NRS‐11, for patients within each respective treatment group (*p* < 0.0001) (Figure 2A). At baseline, pain intensity was similar in both treatment groups, with a mean NRS‐11 score of 6.2 and 6.4 for RCT and pulpotomy‐treated patients, respectively (Figure 2B). However, participants who received RCT were observed to have a significantly greater decrease in pain intensity between baseline and day 3, with a mean NRS of 1.7 (*p* = 0.0034) and from day 3 to day 7, with a mean NRS of 0.7 (*p* = 0.0471), compared with participants treated with pulpotomy, who had mean NRS of 2.4 and 1.2 respectively at the same time points.

**FIGURE 2:**
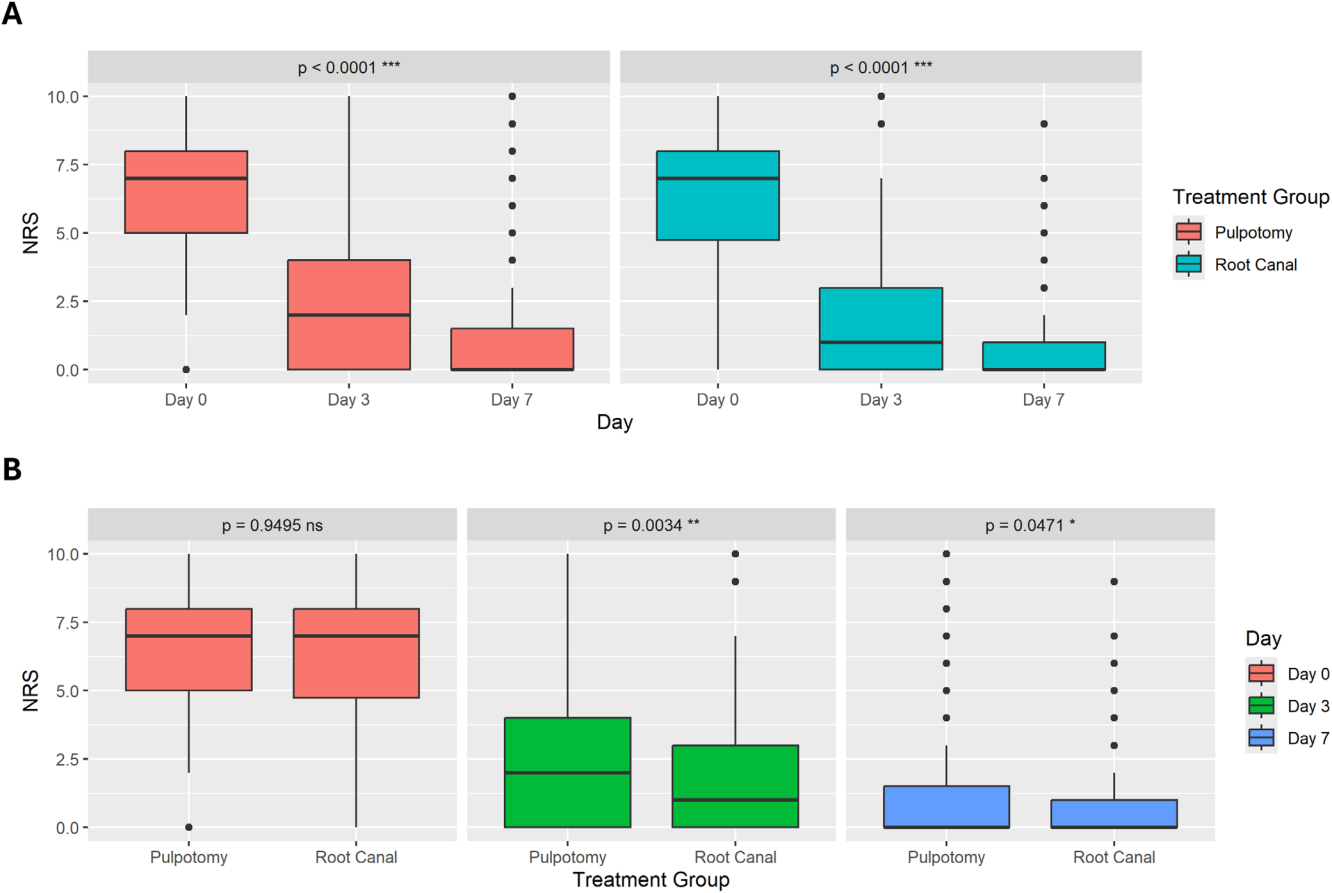
Boxplots demonstrating pain intensity over time according to treatment received (A) and pain intensity according to treatment received over time (B) (*n* = 363). Patients with missing data for the numeric rating scale‐ 11 (NRS) at any time‐point (*n* = 22) were excluded from the analysis. *p*‐values in (A) based on the Friedman test for differences in distribution between more than two paired groups. *p*‐values (B) based on the Mann–Whitney *U* test for differences between two independent groups. A *p*‐value of < 0.05 was considered significant.

### 3.2 Need for Postoperative Analgesic Intake at Day 3 and 7

Analysis of pain relief within treatment groups, as reported by trial participants at each time point, indicated that analgesic use remained high between baseline and day 3 for participants treated with pulpotomy (75/137, 54.7% vs. 70/137, 51.1% for analgesic usage in baseline and day 3; *p* = 0.6025) but significantly reduced between day 3 and day 7 (70/137, 51.1% vs. 27/137, 19.7% for analgesic usage in day 3 and day 7; *p* < 0.0001) (Figure 3A). In contrast, the use of analgesics decreased following RCT, from 49.3% usage (68/138) at baseline to 33.3% (46/138) at day 3 (*p* = 0.0097) and 9.4% (13/138) at day 7 (*p* < 0.0001). In both treatment groups, approximately half the participants who reported on pain relief were taking an analgesic at baseline (Figure 3B). The use of analgesics at day 3 was significantly higher for participants treated with pulpotomy than for those who received RCT (51.1%, 70/137 vs. 33.3%, 46/138; *p* = 0.0042). As noted above, the use of analgesics decreased again between days 3 and 7 for both treatment groups but, at day 7, twice as many patients were still taking pain relief medication after pulpotomy compared with those treated with RCT (19.7%, 27/137 vs. 9.4%, 13/138; *p* = 0.0246).

**FIGURE 3:**
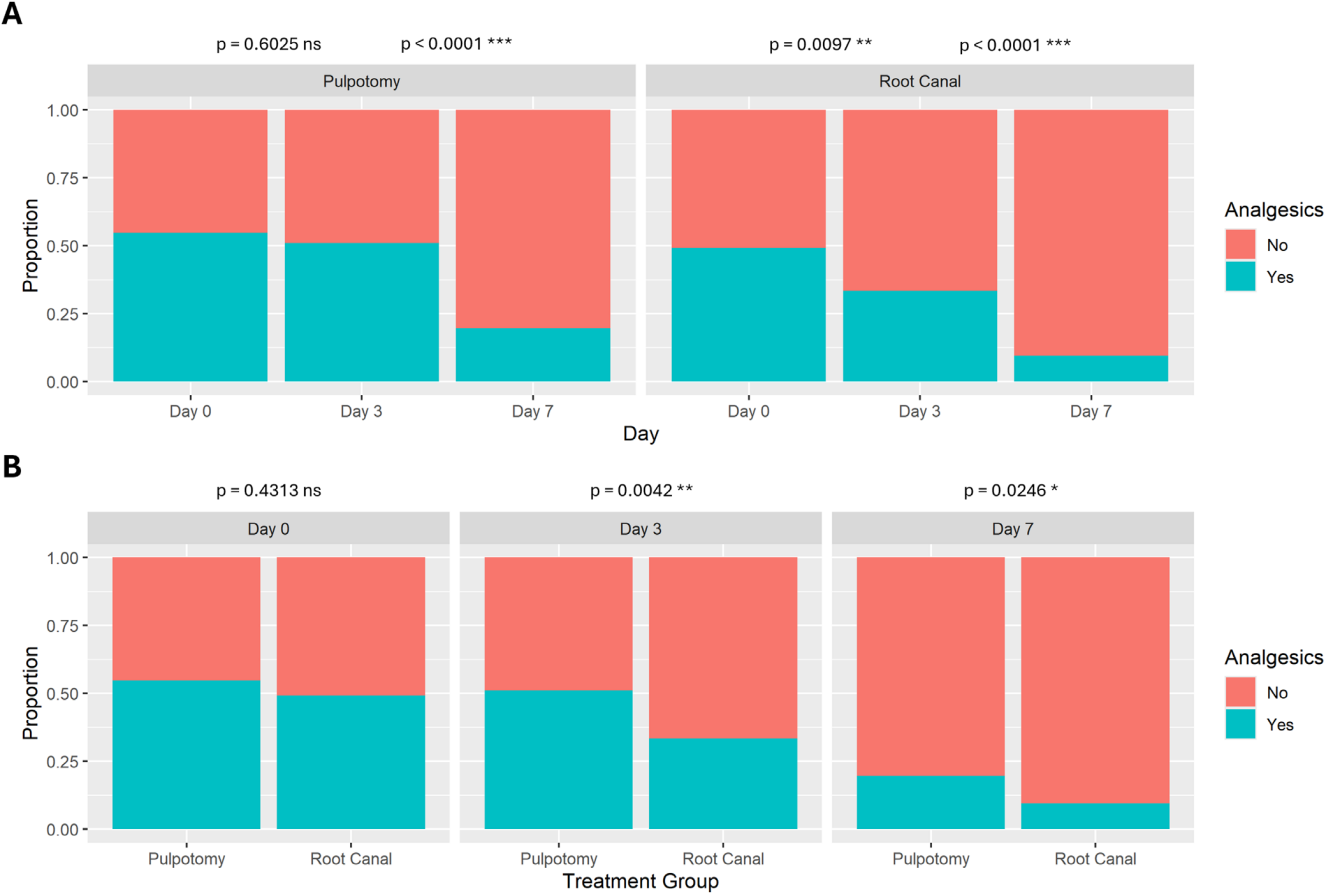
100% stacked bar plots demonstrating the use of analgesics according to treatment received over time (A) and the use of analgesics over time in PROVE participants according to treatment received (B) PROVE participants according to treatment received (*n* = 275). Patients with missing data for analgesics at any time point (*n* = 110) were excluded from this analysis. *p*‐values in (A) were based on McNemar test for association in matched samples. *p*‐values in (B) were based on chi‐squared test for association. A *p*‐value of < 0.05 was considered significant.

### 3.3 Incidence of Early Failure of Pulpotomy Compared With Root Canal Treatment

Early failure (within 4 weeks) was observed in significantly more participants who underwent pulpotomy treatment (10/192; 5.2%) than in those who received RCT (1/193; 0.5%) (*p* = 0.0141) (Figure S1). The most common cause of failure was persistent pain (9/10) and tooth fracture requiring extraction (1/10).

To investigate the possible association of early failure with clinical variables in participants who had pulpotomy, chi‐squared tests for association showed that the tooth being preoperatively TTP (*p* < 0.0001) or a molar (*p* = 0.0001) was associated with early failure and there was also an association with sleep disturbance (*p* = 0.0123) (Table 2). However, these results should be interpreted with caution due to the low number of failure events in the data (10 teeth). For this reason, it was not possible to perform multivariate analysis to rule out any confounding effects.

**TABLE 2:** Association of clinical variables with early failure in pulpotomy‐treated participants.

	Failed (*n* = 10)	Successful (*n* = 182)	*p*
Country			
Egypt	0 (0.0%)	21 (11.5%)	0.1789
India	4 (40.0%)	26 (14.3%)	
Jordan	2 (20.0%)	25 (13.7%)	
Malaysia	0 (0.0%)	22 (12.1%)	
Northern Ireland	3 (30.0%)	27 (14.8%)	
Singapore	0 (0.0%)	18 (9.9%)	
Thailand	1 (10.0%)	23 (12.6%)	
United Arab Emirates	0 (0.0%)	20 (11.0%)	
Gender			
Male	3 (30.0%)	80 (44.0%)	0.5895
Female	7 (70.0%)	102 (56.0%)	
Age	Range: 14–53, Median: 29	Range: 10–78, Median: 31	0.7812
Tooth number			
MO	7 (70.0%)	147 (80.8%)	0.0001
PR	2 (20.0%)	35 (19.2%)	
Missing	1 (10.0%)	0 (0.0%)	
Stimulated or spontaneous pain			
No Pain	0 (0.0%)	6 (3.3%)	0.0884
Spontaneous	2 (20.0%)	95 (52.2%)	
Stimulated	8 (80.0%)	81 (44.5%)	
Tenderness to percussion (TTP)			
0	5 (50.0%)	125 (68.7%)	< 0.0001
1	4 (40.0%)	57 (31.3%)	
Missing	1 (10.0%)	0 (0.0%)	
Use of analgesics			
No	4 (40.0%)	79 (43.4%)	0.9248
Yes	5 (50.0%)	80 (44.0%)	
Missing	1 (10.0%)	23 (12.6%)	
Sleep disturbance			
No	6 (60.0%)	96 (52.7%)	0.0123
Yes	3 (30.0%)	85 (46.7%)	
Missing	1 (10.0%)	1 (0.5%)	
Caries depth			
Deep	1 (10.0%)	50 (27.5%)	0.5053
Extremely deep	5 (50.0%)	88 (48.4%)	
None	0 (0.0%)	2 (1.1%)	
Missing	4 (40.0%)	42 (23.1%)	
Number of tooth surfaces affected			
1	2 (20.0%)	28 (15.4%)	0.2290
2	3 (30.0%)	108 (59.3%)	
3	1 (10.0%)	15 (8.2%)	
4	0 (0.0%)	0 (0.0%)	
Missing	4 (40.0%)	31 (17.0%)	
Dental dam used			
No	1 (10.0%)	6 (3.3%)	0.5324
Yes	9 (90.0%)	175 (96.2%)	
Missing	0 (0.0%)	1 (0.5%)	
Operator			
Endodontist	4 (40.0%)	51 (28.0%)	0.7256
General dental practitioner	3 (30.0%)	36 (19.8%)	
Paediatric dentist	0 (0.0%)	5 (2.7%)	
Post‐graduate dentist	2 (20.0%)	68 (37.4%)	
Missing	1 (10.0%)	22 (12.1%)	

*Note: p*‐values based on chi‐squared tests for association for categorical variables and the Mann–Whitney *U* test for continuous variables. A *p*‐value of < 0.05 was considered significant.

Abbreviations: MO, molar; PR, premolar.

Early failure in participants who underwent pulpotomy was not associated with NRS at baseline (*p* = 0.5083) (Figure S2A) but was significantly associated with time to achieve haemostasis within 10 min (*p* = 0.0006) (Figure S2B). Patients who failed had a mean time to haemostasis of 7.0 min (range 4–10 min) compared with 4.0 min (range 1–10) for those who did not fail.

## 4 Discussion

The concepts of maintaining pulp vitality and using minimally invasive VPTs, such as pulpotomy, gained popularity over the last few decades based on early efficiency studies demonstrating promising success (Cushley et al. 2019). However, in order to develop clinical guidelines to inform clinical practice, the need for evidence of the effectiveness of such interventions has been noted (Duncan, Galler, et al. 2019; AAE Position Statement on Vital Pulp Therapy 2021; Duncan et al. 2023).

In this first report of the multi‐centre PROVE study, the short‐term outcomes of pulpotomy compared with RCT are presented. The results showed that both pulpotomy and RCT provided significant reduction in pain scores at day 3 and 7, but those receiving RCT showed significantly more pain relief than pulpotomy participants at these two time points. Furthermore, analgesic intake was significantly reduced in both groups compared with baseline, but with a greater decrease in the RCT group at day 3 compared with the pulpotomy group. There was also a higher early failure in teeth treated with pulpotomy compared with RCT, which was associated with teeth that were TTP or a molar tooth, pain that disturbed sleep and longer time to achieve pulpal haemostasis after exposure.

These results concur with earlier randomised trials (Asgary and Eghbal 2010; Taha et al. 2023) by highlighting that a significant reduction in pain was evident after either pulpotomy or RCT but also diverge by highlighting a greater reduction in pain for patients receiving RCT. A few clinical trials have compared pulpotomy with RCT and reported on postoperative pain (Asgary and Eghbal 2010; Eghbal et al. 2020; Taha et al. 2023). In contrast to the PROVE findings for postoperative pain, (Asgary and Eghbal 2010) reported lower pain scores for pulpotomy compared with RCT. Their findings indicated that the average time required for pain relief in the RCT group was 36 and 18 h for pulpotomy, which was statistically significant. A later study by the same group (Eghbal et al. 2020), conversely, reported no difference in postoperative pain scores between the two treatment groups.

Although RCT demonstrated statistically lower short‐term pain scores under the conditions of this trial, these findings should be interpreted cautiously. Pulpotomy remains a biologically sound, minimally invasive and clinically feasible treatment option in appropriately selected cases. While between‐group differences in NRS pain scores reached statistical significance, the absolute magnitude of these differences (approximately 0.7–1.2 points) is modest and may not exceed the minimal clinically important difference of 1–2 units reported for pain (Farrar et al. 2001). Consequently, the observed differences may not consistently reach thresholds considered clinically meaningful and the results should therefore be interpreted with caution when inferring clinical superiority. It could also be argued that preoperative analgesic intake may have influenced baseline and early postoperative pain scores by attenuating symptoms prior to treatment. However, randomisation resulted in comparable baseline pain levels between groups, suggesting that any residual effects of analgesics were evenly distributed and unlikely to have biased between‐group comparisons.

In this study, *early failure* was defined as the need for additional clinical intervention within 4 weeks following treatment, most commonly due to persistent or intolerable postoperative pain. This definition was intended to capture clinically meaningful early outcomes; however, early failure may not solely reflect procedural inadequacy. In some cases, it may represent pre‐existing disease severity or limited pulpal reparative capacity that could not be reliably identified preoperatively. The association of failure with the tooth being TTP, nocturnal discomfort pain and prolonged time to achieve haemostasis reported in this study may indicate severe pulpitis not amenable to pulp preservation approach. In concurrence with this, recent studies comparing pulpotomy with RCT have shown that teeth in the pulpotomy group with a diagnosis of severe pulpitis and those with evidence of periapical changes and prolonged time to achieve haemostasis were associated with high postoperative pain scores post treatment (Sarı et al. 2024; Patel et al. 2025).

The diagnostic uncertainty inherent in the clinical assessment of irreversible pulpitis may have contributed to early failure in the pulpotomy group (Donnermeyer et al. 2023). The current diagnostic methods do not always reliably reflect the true histological extent of pulpal inflammation. As a result, some teeth selected for pulpotomy may have had more extensive inflammation to be adequately managed with coronal pulpotomy. Furthermore, the reliance on clinical indicators such as pain characteristics, sensibility testing and absence of periapical pathology may have limited sensitivity and specificity in accurately stratifying disease severity. Future diagnostic classifications based on criteria to define the severity of pulpitis (Wolters et al. 2017; Karrar et al. 2025) and use of objective biomarkers (Karrar et al. 2023) may help with case selection for predicable VPT (Duncan 2022; Karrar et al. 2023). However, until appropriate diagnostic methods are made available, clinicians may use the above features to help with case selection, treatment decision‐making and informed patient consent.

It is important however, to interpret our early failure results with caution because the failure event rate is low (5%). The exclusion of patients in whom haemostasis was not achieved or the pulp was found to be necrotic after randomisation may have contributed to this low failure rate, but this approach is required for good clinical and research practice. At present, no evidence‐based haemostasis time limit has been established to distinguish reliably between reversible and non‐reversible pulpal inflammation. By extending the threshold to 10 min, we sought to include teeth with more advanced inflammatory states while maintaining biological plausibility for healing. It is likely that failure to achieve haemostasis or subsequent early failure, may reflect an inflammatory burden already beyond reparative capacity at the time of treatment, which warrants further investigation.

### 4.1 Strengths and Limitations

The multi‐centre PROVE study was designed to address critical clinical questions across a linked series of randomised trials that would have required much larger resources to complete as a single randomised trial. Using the efficient design, unified protocol and outcome measures, as well as a sufficiently large sample size and obtaining pragmatic data from patients from different geographical locations and with treatments provided in different healthcare systems with different expertise, PROVE has provided the first robust generalisable evidence on the short‐term outcomes of pulpotomy compared with RCT. Although the PROVE study findings on short‐term outcomes didn't concur with reports of some earlier randomised trials, this discrepancy should not be interpreted as a weakness of the current trial. The PROVE study was designed as a multicentre, international, pragmatic trial, incorporating a heterogeneous patient population and operator cohort representative of routine clinical practice. Such a design enhances external validity and may capture variability in outcomes that is not fully reflected in smaller, single‐centre or highly controlled trials. Differences in sample size, geographic distribution, operator experience and protocol implementation may reasonably contribute to variation in observed outcomes. In this context, the present findings should be viewed as complementary to existing evidence, highlighting how treatment effects may differ under broader real‐world conditions. Rather than invalidating prior trials, PROVE results underscore the importance of trial design, setting and protocol harmonisation when interpreting outcomes related to vital pulp therapy.

There are, however, some limitations to our approach. A potential for post‐randomisation bias due to intraoperative exclusions may exist. A true intention‐to‐treat analysis, however, was not possible as no outcome data were collected beyond baseline for participants who were excluded intraoperatively. The analyses were therefore conducted on the per‐protocol population, comprising all participants who commenced their allocated intervention, with exclusions reported as unavoidable intraoperative findings. This approach is consistent with CONSORT, which recognises that post‐randomisation exclusions from the analyses may be acceptable when outcomes cannot be meaningfully assessed and exclusions are unavoidable, predefined and transparently reported (Schulz et al. 2010).

Another limitation of this study is the lack of participant and operator blinding, which was unavoidable due to the clinical nature of the intervention that may increase the risk of performance and detection bias. To mitigate this risk, we standardised postoperative pain data collection using the validated NRS‐11 and the data was analysed with a blinded statistician.

We also recognise the presence of missing data for NRS pain scores and analgesic intake. Missingness primarily resulted from incomplete patient self‐reporting rather than study withdrawal. There was no evidence of systematic loss to follow‐up related to treatment allocation or early failure at baseline. Nonetheless, we acknowledge that cumulative missing data from self‐reported outcomes arising across timepoints may introduce bias, particularly for subjective outcomes such as pain. The low number of failures also makes it difficult to determine the impact of the missing data on the results and precluded the ability to conduct comprehensive meta‐analysis and regression modelling as proposed in the published protocol (El Karim et al. 2023).

The pragmatic trial design may introduce heterogeneity related to operator expertise, but this approach was deliberately chosen to reflect routine clinical practice and improve the generalisability of the findings. However, standardised treatment protocols, operator calibration and uniform peri‐ and postoperative instructions were applied to minimise differential bias between groups and in fact our analysis demonstrated no significant differences in treatment success or early failure rates among the four operator groups.

## 5 Conclusion

The PROVE study demonstrates under the specific conditions of its protocol that effective pain relief up to day 7 was evident after both pulpotomy and RCT in teeth with SIRP and that pain relief was greater for patients receiving RCT. Early failure, although infrequent, was increased after pulpotomy and was significantly associated with features suggestive of severe inflammation including TTP, sleep disturbing pain and prolonged pulpal bleeding time. These findings contribute to an evolving and heterogeneous evidence base, highlighting the need for greater harmonisation across studies and for further research to clarify factors associated with early pulpotomy failure.

## Author Contributions

All the authors have made relevant contributions to the manuscript. All the authors have read and approved the final version of the manuscript.

## Funding

The following funding bodies contributed to the PROVE study: Department of Health in Northern Ireland; British Endodontic Society; Health System Research Institute (HSRI) Grant number 67‐041, Thailand; ORCHID Seed Bridging Grant number 22‐5223‐A0002, Singapore; Deanship of Research and IRB approval (grant number 2019/375), Jordan University of Science and Technology; and Dental Postgraduate Research Grant, Faculty of Dentistry, Universiti Malaya (number UMG0 61E‐2024).

## Ethics Statement

The study was performed in accordance with the ethical standards of the Declaration of Helsinki. All patients gave written informed consent prior to their inclusion in the study. Ethical approval was obtained from respective ethics committees at each institution.

## Conflicts of Interest

The authors declare no conflicts of interest.

## Supporting information


**Figure S1:** One hundred percent of stacked bar plot demonstrating the association with early failure in PROVE participants according to treatment received (*n* = 385). *p*‐value based on chi‐squared test for association. A *p*‐value of < 0.05 was considered significant.
**Figure S2:** Boxplot (A) demonstrating NRS at baseline in pulpotomy‐treated patients according to early failure (*n* = 188). Patients with missing NRS at baseline (*n* = 4) were excluded from this analysis. Boxplot (B) demonstrating the time to haemostasis in pulpotomy‐treated patients according to early failure (*n* = 187). Patients with missing time to haemostasis (*n* = 5) were excluded from this analysis. *p*‐value based on the Mann–Whitney *U* test for differences between two independent groups. A *p*‐value of < 0.05 was considered significant.

## Data Availability

The data that support the findings of this study are available on request from the corresponding author. The data are not publicly available due to privacy or ethical restrictions.
