# Supplementary material for: Short‐Term Outcomes of Full Pulpotomy Compared With Root Canal Treatment for Irreversible Pulpitis: The PROVE Study
Source: Int Endod J. 2026 Jun 29;59(11):2335–45. doi: 10.1111/iej.70208 (PMC13674818; doi:10.1111/iej.70208)
Supplement: Supplementary file 1 [file IEJ-59-2335-s001.docx]

***Supp Fig 1****: 100% stacked bar plot demonstrating the association with early failure in PROVE participants according to treatment received (n=385). P value based on chi-squared test for association. A p value of < 0.05 was considered significant.*

***Supp Fig 2****: Boxplot* ***(A)*** *demonstrating NRS at baseline in pulpotomy-treated patients according to early failure (n=188). Patients with missing NRS at baseline (n=4) were excluded from this analysis. Boxplot* ***(B)*** *demonstrating the time to haemostasis in pulpotomy-treated patients according to early failure (n=187). Patients with missing time to haemostasis (n=5) were excluded from this analysis. P value based on the Mann-Whitney U test for differences between two independent groups. A p value of < 0.05 was considered significant.*
